# Single-cell omics analysis reveals functional diversification of hepatocytes during liver regeneration

**DOI:** 10.1172/jci.insight.141024

**Published:** 2020-11-19

**Authors:** Tianyi Chen, Sehhoon Oh, Simon Gregory, Xiling Shen, Anna Mae Diehl

**Affiliations:** 1 Department of Molecular Genetics and Microbiology, Duke University School of Medicine, Durham, North Carolina, USA.; 2 Department of Medicine and; 3 Department of Neurology, Duke University, Durham, North Carolina, USA.; 4 Duke Molecular Physiology Institute, Duke University School of Medicine, Durham, North Carolina, USA.; 5 Department of Biomedical Engineering, Pratt School of Engineering, Duke University, Durham, North Carolina, USA.

**Keywords:** Hepatology, Molecular biology

## Abstract

Adult liver has enormous regenerative capacity; it can regenerate after losing two-thirds of its mass while sustaining essential metabolic functions. How the liver balances dual demands for increased proliferative activity with maintenance of organ function is unknown but essential to prevent liver failure. Using partial hepatectomy (PHx) in mice to model liver regeneration, we integrated single-cell RNA- and ATAC-Seq to map state transitions in approximately 13,000 hepatocytes at single-cell resolution as livers regenerated, and validated key findings with IHC, to uncover how the organ regenerates hepatocytes while simultaneously fulfilling its vital tissue-specific functions. After PHx, hepatocytes rapidly and transiently diversified into multiple distinct populations with distinct functional bifurcation: some retained the chromatin landscapes and transcriptomes of hepatocytes in undamaged adult livers, whereas others transitioned to acquire chromatin landscapes and transcriptomes of fetal hepatocytes. Injury-related signaling pathways known to be critical for regeneration were activated in transitioning hepatocytes, and the most fetal-like hepatocytes exhibited chromatin landscapes that were enriched with transcription factors regulated by those pathways.

## Abstract 2

Mapping state transitions in hepatocytes at single-cell resolution as livers regenerated revealed bifurcation into adult and fetal-like hepatocyte populations.

## Introduction

Adult liver has enormous regenerative capacity. After acute 70% liver resection (partial hepatectomy [PHx]), residual liver cells proliferate in a highly synchronized manner that allows careful and robust investigation of how the tissue completely restores liver mass and function within days in rodents and within weeks in humans. No other adult organ has such capability and instead mainly replaces dead cells with scar tissue that leads to progressive loss of organ function. Emerging data suggest that the unique regenerative potential of the injured liver is linked to the inherent plasticity of adult liver cells; however, the mechanisms that modulate state transitions in adult liver cells to accomplish regeneration while maintaining vital liver-specific functions are not well understood. Cell plasticity during the regenerative process suggests that epigenetic regulation is critical. In previous studies, reciprocal changes in net chromatin accessibility to major transcriptional regulators of hepatic metabolism and proliferation have been described in repopulating hepatocytes (1). However, because the approaches used in those studies did not resolve effects at the level of individual cells, it remains unclear how the hepatic plasticity program manages to simultaneously maintain critical metabolic functions and regeneration, whether by the same or different hepatocyte populations.

Here, we combine single-cell assay for transposase accessible chromatin with high-throughput sequencing (scATAC-Seq) and RNA-Seq to characterize hepatocyte states and transitions that enable successful regeneration of functional hepatic parenchyma after PHx. Our analysis suggests that hepatocytes increase heterogeneity and bifurcate into distinct, quiescent, metabolism-sustaining states and progenitor-like, proregeneration states that orchestrate successful regeneration of functional parenchyma in injured livers while simultaneously fulfilling life-sustaining metabolic, biosynthetic, and detoxification functions. Our integrated analyses further identified unique regulomes associated with chromatin accessibility and expression changes that are specific to regenerative hepatocyte clusters, providing additional insight into the general mechanisms for defective repair, organ failure, and carcinogenesis.

## Results

### scRNA-Seq reveals heterogeneity of hepatocytes during liver regeneration.

Adult liver cells rarely undergo division during homeostatic conditions. However, after the abrupt and massive regenerative challenge imposed by PHx, most mature hepatocytes quickly reenter the cell cycle and proliferate so that the liver regains its developmental potential and restores its original mass and function (2). Previous bulk transcriptomic analyses of different time points following PHx showed that the proliferative response in hepatocytes peaks sharply around 48 hours (3). However, it is unclear how the hepatocyte population adapts to maintain liver-specific functions despite drastically increasing net replicative activity.

**Figure 1:**
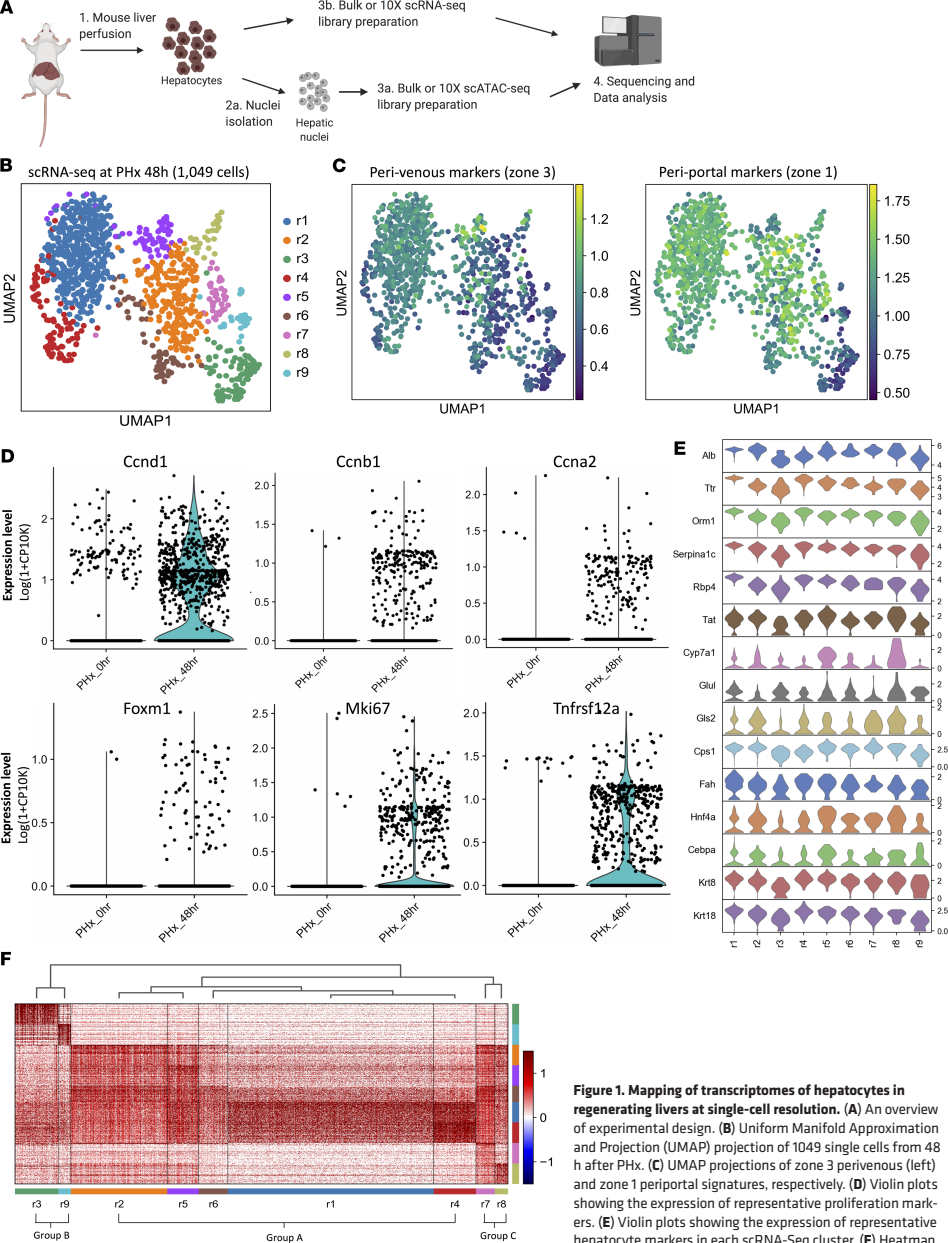
Mapping of transcriptomes of hepatocytes in regenerating livers at single-cell resolution. (**A**) An overview of experimental design. (**B**) Uniform Manifold Approximation and Projection (UMAP) projection of 1049 single cells from 48 h after PHx. (**C**) UMAP projections of zone 3 perivenous (left) and zone 1 periportal signatures, respectively. (**D**) Violin plots showing the expression of representative proliferation markers. (**E**) Violin plots showing the expression of representative hepatocyte markers in each scRNA-Seq cluster. (**F**) Heatmap grouping of cluster signatures.

To address this gap in knowledge, we isolated hepatocytes from 4 healthy adult male mice and from 4 mice that had undergone PHx 48 hours earlier. At each of these 2 time points, RNA or nuclei were extracted from 3 of the hepatocytes preparations and used for either bulk RNA-Seq or bulk ATAC-Seq to generate 3 biological replicates, respectively; one-half of the cells in the fourth hepatocyte preparation at each time point were used to generate single-cell libraries for scRNA-Seq, and nuclei were isolated from the remaining cells for scATAC seq analyses (Figure 1A). First, we assessed the purity of our hepatocyte preparations by using cell signature profiles for hepatocytes, Kupffer cells, and endothelial cells developed by Halpern and colleagues (4) (Supplemental Figure 1A; supplemental material available online with this article; https://doi.org/10.1172/jci.insight.141024DS1) to deconvolute the bulk RNA seq data from each of the 3 biological replicates/time points and demonstrated that 96%–98% of the cells isolated in each replicate were hepatocytes (Supplemental Figure 1B). To confirm that the hepatocyte isolates that were further processed to generate either single cells or single nuclei were pure, we next applied the same single-cell signatures to our single-cell RNA-Seq data. Uniform Manifold Approximation and Projection (UMAP) of data from the single-cell preparations at both time 0 and 48 hours after PHx were consistent with the results of the deconvoluted data from the bulk isolates at these time points and demonstrated that the cells used for single-cell library preparations were almost exclusively hepatocytes based on expression profiles of cell type–specific gene signatures previously validated by others (4) (Supplemental Figure 1, C and D).

The time point 48 hours after PHx is known to fall within the brief window of hepatocyte proliferative activity after this regenerative challenge. Therefore, as proof of principle, we confirmed that our bulk RNA-Seq data (Supplemental Figure 2, A and B) matched that of others who had previously reported that this time point coincides with extensive reparative activity and maximal hepatocyte proliferation (3). We then verified that the global expression profiles from our pooled scRNA-Seq data correlated with that of our bulk RNA-Seq data (*r* = 0.83, Supplemental Figure 2C), indicating the high quality of our single-cell library. After filtering out minimally expressed genes and cells with high mitochondrial content, approximately 1049 cells met the quality metrics, and their transcriptomes were used for subsequent analyses (Supplemental Figure 3A). Unsupervised graph-based clustering (5) identified 9 clusters, which we term r1–r9, each containing between 27 and 438 cells (Figure 1B; Supplemental Figure 3B).

### Zonal differences in hepatocyte gene expression decrease as hepatocytes respond to a regenerative challenge.

In undamaged livers, it is well established that hepatocyte gene expression varies significantly across liver lobules based on portal-to-venous perfusion gradients (4). We suspected PHx might disrupt these zonal gene expression signatures because PHx abruptly and dramatically impacts liver blood flow by acutely removing 70% liver mass. Therefore, initially we used published gene expression signatures of perivenous, mid-zonal, and periportal hepatocytes previously reported by Halpern and colleagues (4) to deconvolute our bulk RNA-Seq data from undamaged livers. Similar to the findings of Halpern et al., we found that relatively small subsets of hepatocytes strictly express only perivenous or periportal signatures, whereas most hepatocytes coexpress perivenous and periportal markers (data not shown). Therefore, we applied a dichotomized signature (perivenous vs. periportal) to our PHx48h scRNA-Seq data set (Supplemental Figure 3C). UMAP projections of those data show that most cells in regenerating livers coexpress the perivenous and periportal signatures (Figure 1C; Supplemental Figure 3D). To determine how transcript expression correlates with protein expression of various markers, we performed IHC on additional mice that were sacrificed either before or after PHx and confirmed that typical protein expression of metabolic genes normally restricted to discrete zones in undamaged livers (e.g., Glul, zone3; Gls2, zone 1), and genes that encode secreted hepatocyte-specific proteins (e.g., albumin, zones 2–3), diminish and/or become more diffuse at 48 hours after PHx (Supplemental Figure 3E). Together, these results demonstrate that global changes in hepatocyte-specific gene expression occur when proliferative activity is maximal in the hepatocyte compartment.

### Hepatocytes segregate into functionally diverse subpopulations after a regenerative challenge.

We then sought to determine if previously characterized changes that occur in hepatocytes after PHx could be detected in our single‑cell experiment by comparing scRNA-Seq data at 48 hours after PHx to scRNA-Seq analysis of primary hepatocytes isolated from a healthy mouse. After stringent filtering, we obtained 1196 hepatocytes from the undamaged liver (Supplemental Figure 3A) and compared their pooled transcriptome to that of hepatocytes from the regenerating liver to assess changes in the expression of genes known to be essential for liver regeneration. As expected, critical cell cycle regulators and other proliferative genes, including Tnfrsf12a, a gene that encodes a receptor required for mouse liver regeneration (6), increased after PHx (Figure 1D). The number of cells expressing genes that promote adult hepatocyte dedifferentiation into bipotent liver progenitors (Yap, Gata6) (7, 8) and mark hepatocyte precursors (AFP) (9) or immature cholangiocytes (e.g., Sox9, Hnf1b) (10, 11) also increased (Supplemental Figure 3F). However, expression of C/EBPα and Esrp2, growth-inhibitory genes induced in terminal hepatocyte development (12, 13), genes specifying hepatocyte-specific metabolic functions (Gls2) (14), and other signature hepatocyte genes (e.g., albumin, HNF4α) (15) remained robust (Supplemental Figure 3G), suggesting that regeneration-related changes in the hepatocyte transcriptome are more heterogeneous than previously suspected (1). Indeed, scRNA-Seq analysis showed that although all regenerative clusters displayed high levels of hepatocyte-specific gene expression (Figure 1E), further hierarchical clustering according to transcriptomic similarities segregated cells into 3 major groups (Figure 1F). Differentially expressed genes were used to characterize the most significant biological signatures within each group. The first major hierarchical branch, group A (composed of regenerative clusters r1, r2, r4, r5, and r6), was associated with small molecule biosynthesis, monocarboxylic acid metabolism, steroid metabolism, and drug metabolism (Supplemental Figure 3H). The second major hierarchical branch, group B (composed of clusters r3 and r9), was associated with high receptor tyrosine kinase signaling, RNA splicing, translational initiation, Rho GTPase effectors, actin cytoskeleton reorganization, and signaling related to endocytosis, phagosomes, and lysosomes (Supplemental Figure 3I). The third major hierarchical branch, group C (composed of clusters r7 and r8), was enriched in pathways involved in cholesterol biosynthesis, lipid homeostasis, bile secretion, complement and coagulation cascades, RNA splicing, and response to ER stress (Supplemental Figure 3J).

**Figure 2:**
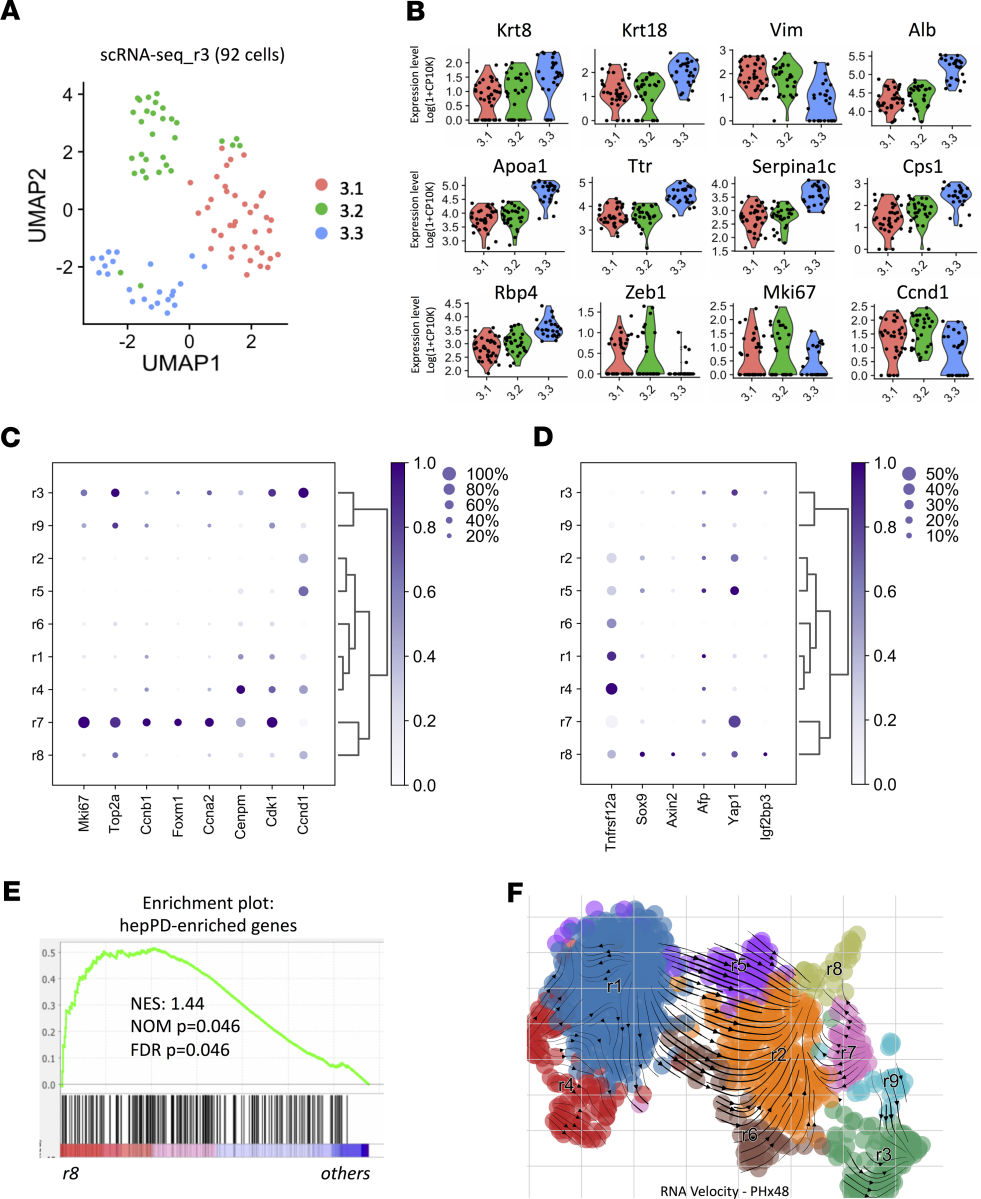
Expression data enables identification of distinct subtypes within each cluster. (**A**) UMAP projection of r3 (*n* = 92 cells). (**B**) Violin plots showing expression of representative markers across subclusters r3.1, r3.2, and r3.3. (**C** and **D**) Dot plot showing the expression of either representative proliferative (**C**) or stem/progenitor markers (**D**) in each cluster. Dot diameter indicates proportion of cells per cluster expressing a given gene; color depicts average expression level of that gene. (**E**) Gene set enrichment analysis (GSEA) of published markers for hepatocytes-derived proliferative ducts (hepPD) shows enrichment in r8 compared with other clusters (r1–r7, r9). (**F**) RNA velocity analysis of hepatocytes at 48 h after PHx.

Pathways involved in regulating morphogenesis were particularly upregulated in regenerative cluster r3 (Supplemental Figure 3I). A more detailed analysis of cluster r3 revealed that this compartment was transcriptionally heterogeneous and could be further divided into 3 subclusters (Figure 2A). Subcluster 3.3 expressed the highest levels of epithelial genes and the lowest levels of mesenchymal markers (Figure 2B). Expression of hepatocyte‑specific markers was also highest in subcluster 3.3. The lowest expression of epithelial markers and hepatocyte-specific genes was in subcluster 3.1, which contained cells that were enriched for proliferative markers, markers of mesenchymal cells, and Zeb1, a transcriptional regulator of epithelial-mesenchymal transitions (EMT). Subcluster 3.2, which was positioned adjacent to relatively mesenchymal subcluster 3.1 on UMAP, showed a hybrid expression pattern that was simultaneously enriched for epithelial and hepatocyte-specific features as well as vimentin. Thus, we concluded that cluster r3 was likely to contain proliferative hepatocytes undergoing EMT. However, overall expression of the hepatocyte gene signature tended to be lower in r3 and r9 than in the other regenerative clusters, and gene expression signatures of liver nonparenchymal cells were stronger in r3 and r9 than in the other clusters (Supplemental Figure 1D). Therefore, we further screened r3 and r9 (and all of the other regenerative clusters) for markers of hepatic stellate cells (HSCs) and portal fibroblasts (PFs) to evaluate potential contamination with these liver- resident mesenchymal type cells. Apart from a rare subpopulation of α-smooth muscle actin (αSMA-expressing) cells in r8, only some of the cells in r3 and r9 expressed markers of HSCs (lecithin retinol acyltransferase, desmin, and αSMA) or PFs (elastin and mesothelin) (Supplemental Figure 4A). In r3, these cells were largely restricted to the most mesenchymal subcluster and further analysis of transcript pairs at the single-cell level confirmed that individual cells that expressed HSC or PF genes uniformly coexpressed some hepatocyte genes, including albumin (Supplemental Figure 4B). A similar analysis of r9 for immune cell markers demonstrated that subpopulations of cells in that cluster coexpressed macrophage and hepatocyte genes at the single-cell level (data not shown). Further research is needed to determine the significance of these relatively rare cells with hybrid gene signatures; however, unless specifically noted, we excluded clusters r3 and r9 from further analysis to avoid bias interpretation of hepatocyte-specific data.

Regenerative cluster r7 was the most proliferative cluster among all hepatocyte subpopulations, and exhibited greater enrichment for proliferative markers than clusters r3 and r9, the 2 next most proliferative clusters (Figure 2C). In contrast, proliferative markers were virtually absent from cluster r8, which expressed markers of biliary-type progenitors (Tnfrsf12a, Sox9) (16–18), hepatocyte progenitors (Axin2, Afp) (9, 19), and multipotent liver stem-like cells (Yap, Igf2bp3) (Figure 2D) (7, 20). Additionally, r8 was significantly enriched for the hepatocyte derived ductal-type progenitor signatures previously identified in chronic injury (21), further supporting the fetal-like nature of r8 (Figure 2E). Given the hierarchical similarities, clusters r7 and r8 likely represent subsets of actively cycling hepatocytes and relatively nonproliferative liver epithelial progenitors, respectively. Taken together, the findings obtained from transcriptomic profiling indicate that during liver regeneration, hepatocytes diversify into fractions that undergo proliferative reprogramming and fractions that maintain essential metabolic responsibilities of the liver.

### Trajectory analysis indicates that functional heterogeneity of hepatocytes in regenerating liver results from dynamic reprogramming of hepatocyte gene expression.

To explore the dynamics of hepatocyte phenotypic transitions in regenerating liver, we performed RNA velocity analysis (22, 23), which leverages ratios of spliced versus unspliced RNA molecules to predict future states of cells. The transitional velocities are reflected by the size of vectors and direction of arrows. Our trajectory analysis (Figure 2F) showed that cells within clusters r1 and r4, which expressed high levels of metabolism‑related genes, were relatively devoid of vectors; cells in clusters r2, r5, r6, and r7 exhibited many long, strong vectors and appeared to mainly undergo rapid transitions toward another distinct state, cluster r8. The progenitive nature of cluster r8 (Figure 2, D and E) and the hybrid epithelial/mesenchymal characteristics of r3 (Figure 2B and Supplemental Figures 4, A and B) suggest that these velocity paths represent real-time reprogramming events in which mature hepatocytes were dedifferentiated into more primitive states during the regenerative process. Notably, r2 and r5 appeared to be the 2 most likely clusters contributing to development of r8 (Figure 2F). Correlation analysis using zonal layer signatures from Halpern’s et al. (4) revealed that r5 was more closely related to the central area, whereas r2 was more similar to cells in mid-zonal-to-portal areas (Supplemental Figure 4C), suggesting that the origin of cells in r8 was not biased toward a specific zone. Rather, hepatocytes all along the central-to-portal axis may have the potential to dedifferentiate and acquire a more fetal-like state.

Furthermore, the vector fields in regenerative hepatocytes revealed complex population kinetics. In particular, we noted a strong directional flow from cluster r1 to cluster r2 that was resolved through clusters r5 and r6, suggesting that clusters r1 and r4 (the cluster most closely related to r1 in our hierarchical cluster analysis) were potential contributors to the inferred dynamics. To further elucidate the functional differences that distinguished clusters r1 and r4 from clusters r2, r5, and r6, we carried out gene set enrichment analysis (GSEA) (24, 25). Gene expression for oxidative phosphorylation, myc targets, adipogenesis, and reactive oxidation pathways were significantly higher in clusters r1 and r4, suggesting that these clusters were heavily enriched with mature, metabolism-focused hepatocytes (Supplemental Figure 4D). In contrast, upregulated genes in clusters r2, r5, and r6 demonstrated significant enrichment for signaling pathways previously shown to control liver regeneration after PHx, including Hedgehog (26), IL6-JAK-STAT3 (27), and TNF-α (28) (Supplemental Figure 4E). Processes known to be regulated by those pathways (e.g., inflammatory responses and EMT) also tended to be enhanced in these clusters, further supporting their involvement in the observed reprogramming events.

### ATAC-Seq reveals heterogeneity of hepatocyte chromatin landscapes during liver regeneration.

**Figure 3:**
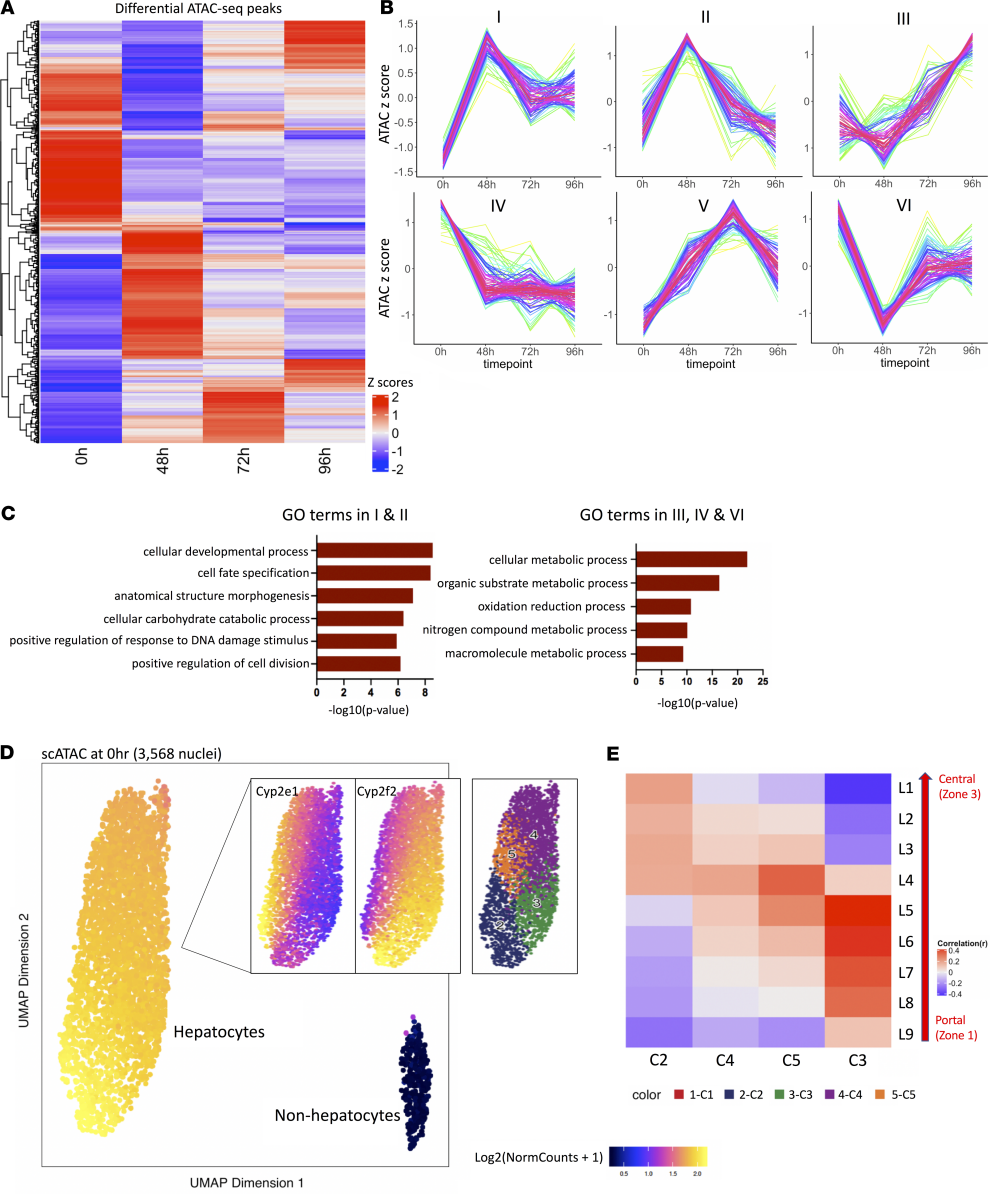
ATAC-Seq analyses during liver regeneration. (**A**) Heatmap displaying significantly altered chromatin accessibility across time (0, 48, 72, and 96 h) after PHx. (**B**) Hierarchical clustering of differentially accessible chromatin in (**B**) revealed 6 modules I–VI. (**C**) Gene Ontogeny (GO) categories enriched in modules I, II (left) and modules III, IV, and VI (right). (**D**) UMAP projection of 3568 single nuclei from undamaged (UD) livers at 0 h. (**E**) Correlation of selected UD clusters with known zonal layers in healthy livers (L1, central; L9, periportal).

The degree to which epigenetic variation occurs in the regenerative process and whether all hepatocytes remodel their chromatin landscapes to enter and exit the proliferative state remain unclear. The latter would indicate very dynamic, global changes in cell fate, and liver-specific functions are generally preserved during the regenerative process. Therefore, to explore temporal changes in the chromatin regulatory landscape that occur during liver regeneration, we generated a time-dependent series of sequencing data from bulk ATAC-Seq by profiling nuclei from hepatocytes that were harvested before and at 48, 72, and 96 hours after PHx (Figure 1A). By comparing each time point following PHx to control undamaged liver (0 h) we identified 551 *cis*-regulatory sequences that were significantly, differentially accessible (DA) in at least one of these time points (Figure 3A). Unsupervised clustering of these 551 regions identified 6 unique modules characterized by their distinct kinetics across the defined time interval (Figure 3B). Five of these six modules (I–IV and VI) were most different from pre-PHx (0 h, undamaged) liver at 48 hours after PHx. Pathway enrichment analysis of the DA regions in each of these modules revealed that they were characterized by unique functions (Supplemental Table 1) (4). For example, modules III, IV, and VI, which demonstrated maximally reduced chromatin accessibility at 48 hours after PHx, were enriched in biological pathways associated with metabolic processes (Figure 3C), in accordance with reports that liver temporarily restricts its typical metabolic functions to meet massive regeneration demands (1). Modules I and II, which demonstrated dramatic increases in chromatin accessibility at 48 hours, showed significant enrichment of Gene Ontology categories linked to embryonic development and tissue morphogenesis (Figure 3C), indicating that hepatocytes at this time point may be involved in a dedifferentiative reprogramming process. However, bulk ATAC-Seq assays only provide an average chromatin profile that is dominated by signals from the most abundant cell populations and, therefore, lack the sensitivity to resolve cellular heterogeneity and subtype specificity. Thus, it was unclear whether all or only a subset of hepatocytes initiated these chromatin changes related to reprogramming.

To address the limitations of bulk assays, we used 10X Genomics to generate single nuclei ATAC-Seq profiles from thousands of individual hepatocytes isolated from healthy adult mice (Figure 1A). To our knowledge, this represents the first single‑cell chromatin accessibility data set in the field. To eliminate low‑quality nuclei, we considered both transcriptional start site enrichment scores and minimal fragments per cell and ultimately retained a data set with 3658 single‑cell ATAC-Seq profiles for downstream analyses (Supplemental Figure 5A). These profiles exhibited a typical ATAC-Seq fragment size distribution, with each library sequenced to an average of 10,562 unique fragments per nucleus and the majority of mapped reads aligning to either intronic or intergenic regions (Supplemental Figure 5, B and C).

To identify epigenetically distinct populations, we used an unbiased clustering strategy adapted from the ArchR (29) package that can efficiently uncover distinct cell populations based on their overall genome-wide similarity. Cells belonging to the same cluster were first pooled to assemble a pseudo-bulk signal profile before peak calling. Using Seurat-implemented clustering algorithms (30, 31), we then generated a chromatin accessibility profile of hepatocytes in undamaged liver (PHx 0 h) and visualized it in UMAP (Figure 3D). To define clusters on this map, we estimated gene score matrices that accounted for both chromatin accessibility across gene bodies and their nearby *cis*-regulatory elements. Our gene-wise annotations revealed 2 distinct populations: a predominant population consisting of hepatocytes and a minor population composed of nonparenchymal cell types, including Kupffer cells and endothelial cells (Supplemental Figure 5, D–F). Hepatocytes were heterogeneous and displayed gradient expression patterns that matched previously defined zonal landmarks (Figure 3E and Supplemental Figure 5G), demonstrating that our scATAC-Seq data from undamaged liver faithfully recapitulated the canonical transcriptional zonation of healthy liver, and that hepatocytes in each zone have a unique chromatin accessibility landscape.

**Figure 4:**
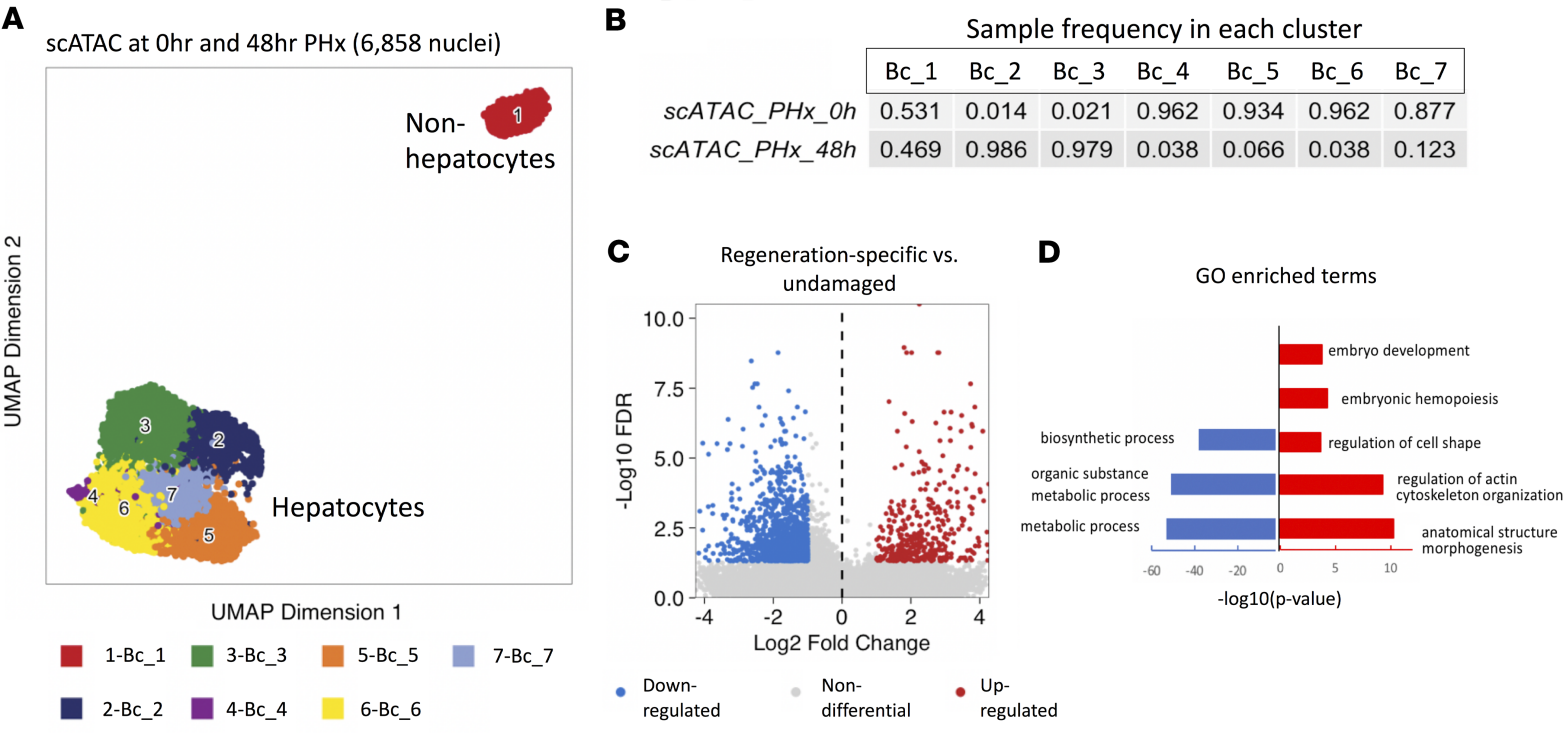
Single-cell ATAC-Seq analyses reveal heterogeneity of hepatocytes in regenerative livers. (**A**) UMAP projection of 6858 single cells across both UD liver (*n* = 3568) and 48 h PHx liver (*n* = 3290). (**B**) Table showing sample frequencies in each cluster. (**C**) Volcano plot showing distribution of chromatin regions with increased (red) or decreased (blue) accessibility in regeneration-specific hepatocytes at 48 h after PHx. (**D**) GO terms either depleted (blue) or enriched (red) in regeneration-specific hepatocytes.

After consolidating the chromatin accessibility framework of hepatocytes in undamaged liver, we used this as a baseline to deconvolute the heterogeneity of hepatocytes at 48 hours after PHx, the time point that coincides with maximal hepatocyte regenerative activity according to our bulk RNA-Seq data (Supplemental Figure 2, A and B) and that of others (3). Using nuclei isolated from hepatocytes at 48 hours after PHx, we generated individual scATAC-Seq libraries and obtained chromatin accessibility data from 3200 single cells. To reveal both shared and distinct molecular features of hepatocytes in regenerating livers at 48 hours after PHx versus undamaged livers (0 h PHx), we combined data sets and analyzed cells from both time points (total 6858 cells) (31, 32). The resulting UMAP projection identified 7 clusters of cells with DA chromatin features (Figure 4A; Supplemental Figure 6, A–D). Among these clusters, clusters Bc_2 and Bc_3 were very distinct in that each cluster was almost exclusively composed of cells from 48-hour PHx (98.6% in Bc_2; 97.9% in Bc_3) (Figure 4B). Therefore, we classified cells from 48-hour PHx livers in these clusters as “regeneration-specific” hepatocytes, whereas the remaining cells obtained at 48 hours were considered to be “undamaged liver-like” hepatocytes. We then grouped cells from 48-hour PHx liver according to their assigned identities (regeneration-specific vs. undamaged liver-like) and performed comparative analysis with hepatocytes in undamaged livers to further resolve the epigenetic diversity. The chromatin accessibility landscape of undamaged liver-like hepatocytes in regenerating livers was similar to that of hepatocytes in undamaged livers, as only 120-DA regions were found (abs|log_2_ fold change| > = 1; *P*_adj_ < 0.05) (Supplemental Figure 6E). In contrast, we identified 1813 DA chromatin regions (398 with increased accessibility; 1415 with decreased accessibility) between regeneration-specific hepatocytes and hepatocytes in undamaged liver (Figure 4C). Peak annotations on these DA regions revealed that embryo development, cytoskeleton organization, and regulation of cell shape were strongly enriched in the regeneration-specific cells, whereas metabolic and biosynthetic processes were depleted (Figure 4D). In line with earlier findings from the bulk assays, these cumulative results indicate that subsets of regenerative hepatocytes remodeled their chromatin structure and underwent adult-to-fetal reprogramming.

### Integrative scATAC-Seq and scRNA-Seq analyses uncovers distinctive chromatin landscapes in fetal-like hepatocyte population.

**Figure 5:**
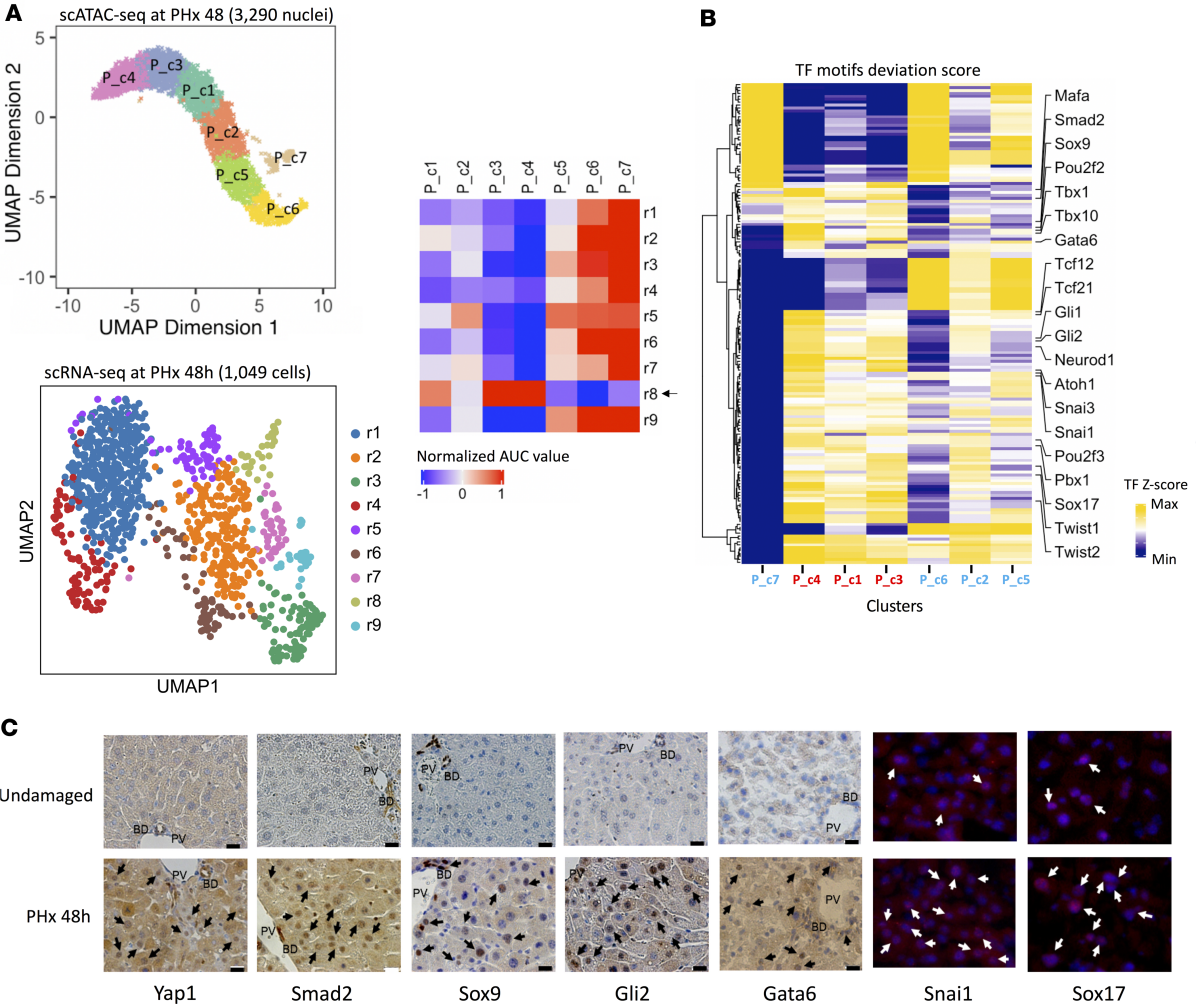
Integrative omics analyses identify transcription factor activities to specific cell subtypes. (**A**) UMAP projection of scATAC-Seq (top left) 48 hours and scRNA-Seq (bottom left) data sets at 48 h after PHx. Correspondence between clusters in these 2 data sets (right). Arrow points to cluster r8. (**B**) Transcription factors (TFs) with significantly enriched activities in P_c1, 3, 4. (**C**) IHC and IF showing cellular localization of representative TFs from P_c1, 3, 4 at high magnification. Black or white arrows indicate positively stained hepatocyte nuclei. Scale bar: 50 μm.

We integrated our independently acquired scATAC-Seq and scRNA-Seq data sets for hepatocytes at 48 hours PHx to jointly reconstitute the hepatic regenerative paths at single-cell resolution (Figure 5A). We used SCENIC (33) an algorithm that can exploit *cis*-regulatory analysis to robustly map the activities of gene regulatory networks in single-cell gene expression, to identify and evaluate potential master regulons (transcription factors [TFs] and their downstream target genes) for each cluster in the scRNA-Seq data (Supplemental Figure 7A). Next, we developed a peak set signature for each scRNA-Seq cluster (r1–r9) using genomic intervals identified from our scATAC-Seq data, of which the peak coordinates had direct overlap with the gene body of each component of top regulons. Last, using cisTopic algorithms (34) we established a link between clusters found in scATAC-Seq data and the derived peak signatures from matching scRNA-Seq data by scoring their respective enrichment with regard to the probabilistic distribution of ranked regions in each cell. Notably, scATAC-Seq clusters P_c1, P_c3, and P_c4 were strongly and exclusively enriched for chromatin features inferred from r8, indicating that these 3 scATAC-Seq clusters of DA chromatins corresponded to the specific chromatin profile of r8 (Figure 5A).

After linking the transcriptomic program of r8 to its chromatin landscape using our ATAC-Seq data set, we next aimed to uncover regulatory factors acting upstream and associate specific TFs to the selected cellular outcomes. Using chromVAR (35) on the scATAC-Seq data from 48-hour PHx to assess and quantify the differential measurement within peaks sharing the same motif, we identified TFs of several families that were likely responsible for the fetal-like state of r8 (Figure 5B). Among these factors, Smad 2, Snai1, and the Hedgehog pathway target Gli2 are transcriptional regulators of EMT, a critical mechanism for cell state transitions during embryogenesis (36). Enrichment for Gata6, Sox9, and Sox17 further supports the stem/progenitor-like characteristics of regenerative cluster r8 (10, 11). Notably, Sox17 marks endodermal progenitors that differentiate into either pancreatic or liver epithelial cells depending on Hedgehog signaling (37), and activation of TFs involved in pancreas development, including Mafa, Pbx1, and Neurod1 (10, 38) was evident in addition to enrichment for Gli2.

We next performed IHC to determine whether any of the 6 aforementioned fetal liver cell-associated TFs accumulate in hepatocyte nuclei at 48 hours after PHx (Figure 5C and Supplemental Figure 7B). Staining for Yap1 was done as a positive control since Yap1 is a stem/progenitor cell-associated transcription cofactor that induces adult hepatocytes to differentiate into a stem-like state when constitutively activated (7, 39). We noted increased hepatocyte nuclear accumulation of each of the 7 fetal state-associated proteins in regenerating liver compared with undamaged liver, in which we observed negligible hepatocyte nuclear staining. After PHx, fetal TF accumulation was heterogeneous and hepatocytes that were positively stained tended to be most abundant periportally (i.e., in zone 1). Isolated ductal cells in portal tracts also expressed some of these markers. Closer inspection of the scRNA-Seq data (Figure 2D) confirmed that expression of Sox9, a marker of ductal cells and small periportal hepatocytes derived from dedifferentiated hepatocytes (21), was greatest in cluster r8 and detectable in r2 and r5, clusters that give rise to r8 in the transition analysis (Figure 2F). Sox9 is induced by Gli2; Gli2 also transactivates Atoh1, a TF that is required for genesis of the primary cilia (40), an obligate structure for canonical Hedgehog signaling (41). Primary cilia are absent in mature hepatocytes but present in liver progenitors that express ductal markers (42). Similar to Sox9 and Gli2, Atoh1 chromatin accessibility was uniquely increased in r8 (Figure 5B). Cyp7a1, which encodes the rate‑limiting enzyme in bile acid synthesis, was also highly expressed in r8 (Figure 1E). Further, GSEA demonstrated that expression of genes involved in bile secretion was enriched in r8 (Supplemental Figure 3I). In aggregate, these findings indicate that 48 hours after PHx, regenerative cluster 8 includes bipotent progenitor cells that are known to emerge in adult livers following other acute or chronic injuries (43) and, more importantly, identify epigenetic regulators of the state change that underlies their outgrowth. Taken together, these data indicate that processes reminiscent of fetal development become reactivated in a subpopulation of hepatocytes in regenerating adult livers.

### Sequential analyses reveal transience of fetal cell state after PHx.

**Figure 6:**
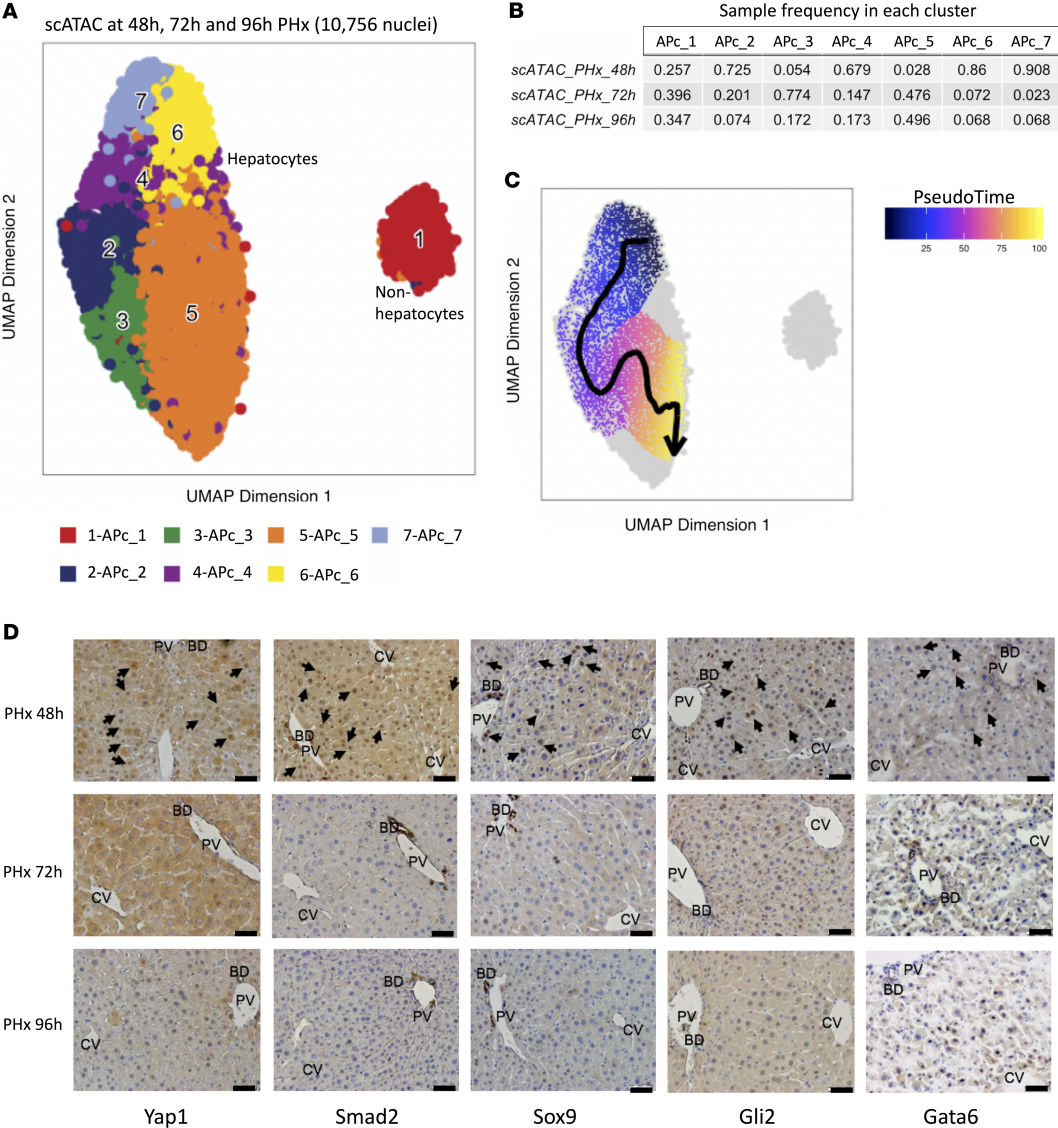
Sequential analyses reveal transience of fetal cell state after PHx. (**A**) UMAP projection of 10,756 single nuclei across 48, 72, and 96 h PHx. (**B**) Proportion of samples in each cluster. (**C**) A UMAP pseudotime trajectory for 10,756 nuclei data sets. Data are color-coded based on the inferred pseudotime spectrum. (**D**) Low-magnification images of TFs that were localized in high-magnification images of PHx 48 h liver sections in Figure 5C. Liver sections from representative mice that were sacrificed at either 48, 72, or 96 h after PHx are shown here. Black or white arrows indicate positively stained hepatocyte nuclei. Scale bar: 20 μm.

To determine how long the fetal-like hepatocytes persisted after an acute regenerative challenge was imposed, we performed scATAC-Seq on freshly isolated hepatocyte nuclei from mice at 2 additional time points, 72 and 96 hours after PHx. Combined analyses of a total of 10,756 nuclei from 48, 72, and 96 hours revealed 7 clusters designated as APc_1-7 (Figure 6A and Supplemental Figure 8, A–C) (30, 31). Cells from different time points were mixed well, and no batch effect was detected, although relative contribution to each cluster was time point dependent (Figure 6B and Supplemental Figure 8D). Pseudotime ordering on the collective data reconstructed the developmental trajectory that started with the fetal state of hepatocytes at 48 hours and returned to mature hepatocytes by 96 hours (Figure 6C and Supplemental Figure 8E) (29). We next performed IHC to confirm that disappearance of the fetal hepatocyte ATAC-Seq “signature” was associated with loss of the 7 representative fetal TFs in liver sections acquired at these later time points (Figure 6D and Supplemental Figure 7B). Staining for each factor at 72 and 96 hours was less than at 48 hours and approached the nearly undetectable levels observed in undamaged liver by 96 hours. Taken together, our analyses indicate that liver damage initiated changes in the chromatin landscape of some adult hepatocytes, which enable their transition into a fetal-like state, coincide with the peak of the proliferative phase and disappear once liver mass is restored.

## Discussion

Tissue injury is an inevitable consequence of life and leads to organ failure if not repaired effectively. The tremendous regenerative capabilities of adult liver distinguish it from other vital organs. Hence, delineating how damaged livers maintain vital functions as surviving hepatocytes regenerate will identify targets to optimize recovery from injury in all organs. Hepatocytes are responsible for maintaining liver-specific functions, while undergoing dramatic switches in proliferative activity, so that injured livers regenerate efficiently and then stop growing once lost liver mass is restored. Our study revealed unexpected heterogeneity in hepatocyte responses to the massive acute regenerative challenge imposed by PHx. Surprisingly, even when hepatocyte proliferative activity was maximal, a significant portion of the residual hepatocytes mostly retained the chromatin landscape of healthy, uninjured hepatocytes and remained preoccupied with fulfilling the essential metabolic responsibilities of the organ. The other larger population of hepatocytes sorted into subpopulations that variably suppressed chromatin accessibility of genes performing adult hepatocyte metabolic functions but generally increased the chromatin accessibility of genes involved in liver development, suggesting that most hepatocytes were undergoing adult-to-fetal reversion to become more proliferative in order to regenerate the resected liver mass. These changes in cell state were dynamic and disappeared once liver mass was restored. Deconvoluting the epigenetic mechanisms that orchestrate and coordinate these complex cell state transitions during an effective regenerative response will identify targets that can be manipulated to correct dysregulated regenerative responses that underlie the pathogenesis of liver failure and cancer.

All of the multiple cell types in adult livers participate in the regenerative process (2). This complicates attempts to map cell-autonomous mechanisms and cell-to-cell interactions that orchestrate successful regeneration of injured livers. The characterization of hepatocytes in healthy livers by single‑cell analytical approaches (4, 44) provides a template for comparison with hepatocytes in injured livers, thus enabling a deeper understanding of the adaptive mechanisms that hepatocyte populations deploy to survive and recover from injury. Our analysis confirms evidence that the perfusion-dependent zonality of liver functions in healthy liver (45) is lost in injured livers (46) and reveals that dynamic genome-wide epigenetic mechanisms are used to disperse these functions, thereby minimizing disruption of systemic metabolic homeostasis while freeing hepatocytes to become more proliferative. The hepatocyte population in regenerating livers remains highly heterogeneous, however, as evidenced by differential chromatin accessibility signatures in hepatocyte subpopulations at a given time after PHx.

Forty‑eight hours after PHx, when hepatocyte proliferative activity was maximal, we identified a small population of hepatocytes that were relatively fetal based on their chromatin landscape and gene expression profiles. This fetal-like state was transient and not apparent 48 hours later, when proliferative activity had subsided and liver mass was nearly recovered. Nevertheless, trajectory analysis at the 48-hour time point after PHx demonstrated that about two-thirds of the more mature hepatocytes were in the midst of adult-to-fetal reversion, which suggests that this state change is critical for an effective regenerative response. GSEA demonstrated that transitioning hepatocyte populations were enriched for critical signaling pathways for liver regeneration after PHx, including TNF-α (28), IL6-JAK-STAT3, and Hedgehog (26, 47). Further, single nuclear ATAC-Seq analysis demonstrated that chromatin in the most fetal-like liver cell population was more accessible to various TFs that are regulated by the aforementioned signaling pathways during development, and IHC confirmed nuclear accumulation of these TFs in subpopulations of hepatocytes. Together, these data indicate that hepatocytes rapidly transition into and out of their regenerative state in injured adult livers by mobilizing epigenetic mechanisms that fetal liver cells use to modulate state transitions during liver development (48, 49). Although this conclusion challenges previous reports that hepatocytes are incapable of undergoing EMT in situ (50), it is consistent with proof that these same TFs interact to constrain hepatocyte differentiation and maintain proliferation in fetal liver cells (51, 52) and bulk analyses of RNA and liver tissues that demonstrate transcriptional regulators of differentiation and proliferation are reciprocally regulated after PHx (1). Importantly, this study reveals regulomes that specify different hepatocyte states and orchestrate state transitions during a regenerative response that engages at least two-thirds of the hepatocytes in the liver remnant. These discoveries complement and extend recent lineage tracing evidence that describes the potential of many adult hepatocytes to reacquire stem cell-like properties in order to regenerate hepatocytes during less massive, but more repetitive, chronic liver injuries (53).

In summary, we combined single nuclear ATAC-Seq, single-cell RNA-Seq, and IHC to analyze hepatocytes at single‑cell resolution in regenerating mouse livers after PHx. Our findings support the concept that many adult hepatocytes can become facultative stem cells and reveal the heterogeneous and dynamic nature of regenerative responses that naturally occur in the hepatocyte compartment when adult liver is abruptly confronted by a finite, but massive, regenerative challenge. Large groups of hepatocytes undergo an adult-to-fetal reversion whereby they transition into less epithelial‑like and more immature cells to become regenerative, whereas other hepatocytes reprogram to maintain vital liver-specific functions despite the massive acute loss of mature hepatocytes. This enormous plasticity is regulated by dynamic epigenetic mechanisms that engage specific cytokine- and morphogen-initiated signaling pathways to differentially modulate the chromatin landscape of hepatocytes in the liver remnant. These findings provide a platform for future research to identify and map the cell-intrinsic and -extrinsic factors that enable signaling diversity. Future studies that clarify the method by which hepatocytes reverse regenerative adaptations to resume their preinjury phenotypes will inform development of novel approaches to prevent and treat outcomes of defective liver repair, including liver failure and cancer.

## Methods

### Animal studies.

Adult male C57BL/6J mice (The Jackson Laboratory) were housed in a barrier facility on a 12:12-hour light/dark cycle with free access to water and standard chow (Purina 5053). At 9–10 weeks of age, PHx was performed (26). Mice were sacrificed at 0, 48, 72, or 96 hours after PHx to obtain either liver tissue (16 mice, *n* = 4 per time point) or primary hepatocytes (16 mice, *n* = 4 per time point). Primary hepatocytes were isolated using a 2-step collagenase perfusion method (54).

### scRNA-Seq and analysis.

Freshly isolated hepatocytes were washed and resuspended in 0.04% UltraPure BSA and counted using the automated cell counter. GEM generation, after GEM-RT cleanup, cDNA amplification, and library construction were performed following 10X Genomics Single Cell 3′ v2 chemistry. Cell Ranger from 10X Genomics was used for initial data processing. Cell-by-gene expression matrices were generated with valid cell barcodes. Individual cells with high mitochondrial contents and possible doublets were further excluded from the downstream analysis. Unsupervised clustering was performed using Scanpy (5) with an appropriate resolution and visualized in UMAP. Cell type–specific markers for hepatocytes, Kupffer cells, and endothelial cells were generated according to Halpern and colleagues (4). We assigned each cell a corresponding score based on its expression of these markers. Pathways enriched in specific clusters were resolved using the GSEA software from the Broad Institute (24, 25). RNA velocity was performed with scVelo (23) using a likelihood-based dynamic model. The splicing kinetics were then resolved and embedded as arrow vectors onto the preexisting clustering layout.

### ATAC-Seq and analysis.

Nuclei were extracted from freshly isolated hepatocytes as previously described (1). Briefly, cells were added into a douncer with prechilled buffer (10 mM Tris-HCl, pH 7.5, 2 mM MgCl2, and 3mM CaCl2) and dounced 15–20 times with a pestle before centrifuging at 50*g* for 5 minutes at 4°C. The supernatant was removed, 1 mL buffer plus 10% glycerol was added to resuspend the cell pellet, then suspensions were incubated on ice for 5 minutes before centrifugation (400*g* for 5 minutes). Nuclei quality was assessed under the microscope, concentration was determined using an automated cell counter, and isolates of 50,000 nuclei/time point were immediately used to generate ATAC-Seq libraries according to a previously published protocol (55). DNA fragments were PCR amplified for a total of 10 cycles. The resulting libraries were purified, size selected using Agencourt AMPure XP beads, and sequenced with an Illumina HiSeq 2500. Sequenced libraries were analyzed with the previously published ATAC-Seq pipeline (https://github.com/ENCODE-DCC/atac-seq-pipeline). Reads were trimmed and aligned to the mm10 reference genome with Bowtie2 (56). Aligned bam files were then subject to removal of PCR duplicates and peak calling by MACS2 (57). Time course analysis was performed using TCseq (https://www.bioconductor.org/packages/release/bioc/html/TCseq.html) with default settings. Significant chromatin features were selected based on both log_2_ fold change > 0.5 and adjusted *P* < 0.05.

### scATAC-Seq and analysis.

Library preparation was performed according to the 10X Chromium Single Cell ATAC-Seq User Guide. Quality was assessed using Agilent DNA tape screen assay. Libraries were then pooled and sequenced using Illumina NovaSeq platform with the goal of reaching saturation or 25,000 unique reads per nuclei on average. Cell Ranger was used for the initial alignment, duplicates removal, and fragments counting. ArchR (29) was used for obtaining a robust clustering on nuclei. To annotate clusters, Cicero (58) was performed to estimate gene activity scores, and the resulting matrices were used to further develop correlation analysis with the previously identified mouse zonal markers (4, 59). We selected 89 genes, which were shown to display strong portal-to-central variation by MacParland and colleagues (59). Pearson correlation coefficient was calculated using *z* scores across all 89 genes to associate the hepatocyte nuclei clusters with portal-to-central layers of mouse liver cells (L1–L9). ChromVAR were used for TF motif enrichment analysis (34, 35).

### Combined scRNA- and scATAC-Seq analysis.

scRNA-Seq and scATAC-Seq data sets were generated from hepatocytes that were isolated from a mouse at 48 hours after PHx. To establish correlations between these 2 data sets, we first used GENIE3 (33, 60) to correlate potential regulatory targets with transcription factors based on their coexpression profiles. For each of the identified target genes, promoter sequences were retrieved from mouse *mm10* genomes and used for motif enrichment analysis (61). Based on the results, target pools were further pruned to retain only target genes with promoters that had binding motifs for the corresponding TF. AUCell (62) was then used to score the activity of each regulon (TF plus its target genes) on a single-cell basis, and the average scores were calculated for cells within defined clusters. Based on the AUCell scores, a list of ranked TF regulons was generated from each cluster r1–r9, respectively (Supplemental Figure 7A). The top-ranked TF regulons represent potential master regulators of cell phenotypes (33). Information obtained from the top 5 regulons in each cluster was then transformed into signature sets (34). In brief, accessible chromatin regions identified from scATAC-Seq data at PHx 48 hours were taken as input to look for the genomic intervals that were located either within or near the gene bodies of TFs and their targets (63, 64). The output was a set of peaks associated with each regulon and for regulons belonging to the same cluster, composite peaks were merged to develop a final consensus feature set for each cluster. Binary accessibility matrix was generated from scATAC-Seq data as input for Latent Dirichlet Allocation and model selection (65). Then, the probability distribution of each region to a cell was estimated (34). These distributions were in turn used to calculate Pearson correlation coefficient between identified scATAC-Seq clusters and the transformed peak set signatures.

### Data availability.

All NGS sequencing data in this manuscript are available at NCBI GEO (accession GSE158864 [RNA-Seq], GSE158865 [ATAC-Seq], GSE158866 [scRNA-Seq], and GSE158873 [scATAC-Seq]).

### Statistics.

All statistics were calculated based on independent replicates. Statistical significance was determined by 2-tailed *t* test unless otherwise stated in figure legends. *P* values of less than 0.05 were considered significant.

### Animal research.

Animal care and surgical procedures were conducted in compliance with an approved Duke University IACUC protocol and the *Guide for the Care and Use of Laboratory Animals* (National Academies Press, 2011).

## Author contributions

TC, SHO, and SG performed the experiments. AMD designed the experiments. TC and AMD conducted the data analyses. TC and AMD wrote the manuscript. XLS edited the manuscript.

## Supplementary Material

supplemental Table 1

## Version 1 11/19/2020

Electronic publication
